# Cardiac Engraftment of Genetically-Selected Parthenogenetic Stem Cell-Derived Cardiomyocytes

**DOI:** 10.1371/journal.pone.0131511

**Published:** 2015-06-25

**Authors:** Tao Yang, Michael Rubart, Mark H. Soonpaa, Michael Didié, Peter Christalla, Wolfram-Hubertus Zimmermann, Loren J. Field

**Affiliations:** 1 Research Center for Translational Medicine, Shanghai East Hospital, Tongji University School of Medicine, Shanghai, China; 2 Riley Heart Research Center, Herman B Wells Center for Pediatric Research, Indiana University School of Medicine, Indianapolis, Indiana, United States of America; 3 Krannert Institute of Cardiology, Indiana University School of Medicine, Indianapolis, Indiana, United States of America; 4 Institute of Pharmacology, University Medical Center Göttingen, Göttingen, Germany; 5 DZHK (German Center for Cardiovascular Research), partner site Göttingen, Göttingen, Germany; Academia Sinica, TAIWAN

## Abstract

Parthenogenetic stem cells (PSCs) are a promising candidate donor for cell therapy applications. Similar to embryonic stem cells (ESCs) and induced pluripotent stem cells (iPSCs), PSCs exhibit self-renewing capacity and clonogenic proliferation *in vitro*. PSCs exhibit largely haploidentical genotype, and as such may constitute an attractive population for allogenic applications. In this study, PSCs isolated from transgenic mice carrying a cardiomyocyte-restricted reporter transgene to permit tracking of donor cells were genetically modified to carry a cardiomyocyte-restricted aminoglycoside phosphotransferase expression cassette (MHC-neo^r^/pGK-hygro^r^) to permit the generation of highly enriched cardiomyocyte cultures from spontaneously differentiating PSCs by simple selection with the neomycin analogue G148. Following engraftment into isogenic recipient hearts, the selected cardiomyocytes formed a functional syncytium with the host myocardium as evidenced by the presence of entrained intracellular calcium transients. These cells thus constitute a potential source of therapeutic donor cells.

## Introduction

Acute myocardial infarction commonly results in the loss of large numbers of cardiomyocytes. Considerable effort has been invested in developing strategies to repair myocardial damage, including the direct injection of donor cardiomyocytes [1,2] as well as the generation and implantation of bioengineered patches, including two dimensional sheets of cells [3] as well as three dimensional constructs [4]. Initial proof of concept studies utilized fetal cardiomyocytes [1], which were followed shortly by embryonic stem cell (ESC)-derived cardiomyocytes [5]. More recently, donor human ESC-derived cardiomyocytes were shown to functionally engraft monkey hearts with small experimentally induced infarcts. Induced pluripotent stem cells (iPSCs) also give rise to functional cardiomyocytes which can be successfully engrafted into donor hearts [6]. Thus, there is mounting evidence that delivery of exogenous cardiomyocyte donor cells can successfully engraft, and in some cases, improve function in damaged hearts.

Chemical stimulation of mammalian ova can give rise to diploid cells which are capable of forming blastocysts in vitro [7]. When cultured under appropriate conditions, these blastocysts can give rise to ESC-like cells designated parthenogenetic stem cells (PSCs). We have recently shown that PSCs give rise to bona fide cardiomyocytes following delivery into host blastocysts, and following either spontaneous or cytokine-directed differentiation in vitro [8]. Moreover, PSC-derived cardiomyocytes derived via directed differentiation can stably engraft following intra-cardiac injection, as well as form functional three dimensional tissue constructs in vitro which survive following surgical attachment to damaged hearts [8]. Because PSCs exhibit a largely haploidentical genotype, it is conceivable that a limited panel of donor cells would be sufficient for “off the shelf” allogenic applications, a highly desirable characteristic for potential clinical application.

We have previously shown that highly enriched populations of cardiomyocytes can be readily generated using ESCs carrying a transgene comprised of the lineage-restricted cardiac myosin heavy chain (MHC) promoter and sequences encoding aminoglycoside phosphotransferase [5]. Treatment of spontaneously differentiating cultures of ESCs carrying this cardiomyocyte selection cassette with the neomycin analogue G418 gave rise to cultures comprised of >99% cardiomyocytes. When amplified in bioreactor, 100s of millions of cells were readily generated [9,10]. In this study, PSCs carrying a cardiomyocyte-restricted enhanced green fluorescence protein (EGFP) reporter transgene as well as the cardiomyocyte selection cassette were employed to demonstrate that highly purified cardiomyocyte cultures could readily be generated. Importantly, these cells formed a functional syncytium with the host myocardium following engraftment into adult recipient hearts.

## Materials and Methods

### PSC culture

We used PSCs (A3 line; generated by M.D. and P.C. in the Zimmermann lab) from mice carrying an MHC-EGFP reporter transgene [8]. These cells were derived from [C57Bl/6J x DBA/2J]F1 mice. The MHC-EGFP reporter targets EGFP expression specifically to cardiomyocytes [11]. The PSC cells were maintained on freshly prepared STO feeder layer in maintenance medium consisting of high glucose DMEM, supplemented with 15% fetal bovine serum (FBS; Invitrogen, Carlsbad, CA, USA), 100 U/ml LIF, 2 mM L-glutamine, 1% non-essential amino acid (NEAA), 0.1 mM β-mercaptoethanol, 1% sodium pyruvate (Sigma, St. Louis, MO, USA), 25 mM HEPES (Sigma), 100 U/ml penicillin and 100 μg/ml streptomycin. To facilitate collection of PSCs for downstream studies, a feeder-free culture condition was optimized, i.e., STO cell-conditioned maintenance medium (CM) was collected and applied to PSC cultures after dilution with fresh maintenance medium at different ratios (25%, 50%, 75% and no dilution designated to 100%; data derived from four individual experiments of 12 replicate studies).

### Transfection of MHC-EGFP PSCs with the transgene MHC-neo^r^/pGK-hygro^r^

A bicistronic cassette comprising an α-cardiac myosin heavy chain-aminoglycoside phosphotransferase (MHC-neo^r^) and a phosphoglycerate kinase (pGK)-hygromycin resistant transgene (pGK-hygro^r^) on a common pBM20 vector backbone (Boehringer Mannheim, Indianapolis, IN, USA) was described previously [5]. This construct, designated *MHC-neo* ^*r*^ */pGK-hygro* ^*r*^ was introduced into MHC-EGFP PSCs via electroporation. To optimize the electroporation voltage, initial studies applied a constitutively expressed *ds-Red* transgene (which could be visualized via epifluorescence) to PSCs. Briefly, PSCs at 1 × 10^7^ in 1 ml 25 mM HEPES were electroporated in the presence of 25 ng *ds-Red transgene* (Bio-Rad MicroPulser, Hercules, CA, USA). The voltage for electroporation was optimized in terms of transfection efficiency (as reflected by percent of red cells versus all attached cells) and cell viability (data derived from four individual experiments of 8 replicate studies). Subsequently, PSCs at 1 × 10^7^ in 1 ml 25 mM HEPES were electroporated with 25 ng *MHC-neo* ^*r*^ */pGK-hygro* ^*r*^ under the optimized voltage. Three days after electroporation, PSCs were selected by hygromycin at 200 μg/ml in optimally diluted STO-CM for 1 week, and independent lineages were derived.

### Polymerase chain reaction (PCR) and Reverse Transcription-PCR (RT-PCR)

PCR analysis was used to confirm the presence of MHC-EGFP and MHC-neo^r^/pGK-hygro^r^ transgenes and RT-PCR analysis to confirm expression of stemness genes. Total DNA and RNA were extracted by the TRIzol Reagent (Invitrogen), according to the manufacturer’s instructions. The extracted DNA or cDNA generated via reverse transcription reaction (RNeasy extraction mini kit; Qiagen, Hilden, Germany) were subjected to PCR analysis as indicated in Table 1.

**Table 1:** Primer sequences and product sizes of targeted genes

Target genes	Forward (from 5´ to 3´)	Reverse (from 5´ to 3´)	Size (bp)
GAPDH	GTGCTGAGTATGTCGTGGAG	ATGCAGGGATGATGTTCTG	357
Oct-4	GGAAAGCAACTCAGAGGGAA	TGTACCCCAAGGTGATCCTC	173
Nanog	AGGGTCTGCTACTGAGATGCTCTG	CAACCACTGGTTTTTCTGCCACCG	363
Nkx2.5	CAAGTGCTCTCCTGCTTTCC	CTGTCGCTTGCACTTGTAGC	349
GATA-4	CTCCTACTCCAGCCCCTACC	TAGTCTGGCAGTTGGCACAG	359
MHC	AGTTCGACAAGATCGAGGACA	GTGCCCTTGTTTGCATTAGG	400
MLC-2v	TTCTCAACGCATTCAAGGTG	TTCAGGGCTCAGTCCTTCTC	214
MHC-eGFP	ATCGAGCTGAAGGGCATCGACTTCAAGGAG	CTCCAGCAGGACCATGTGATCGCGCTTCTC	299
MHC-neo^r^/pGK-hygro^r^	TCCTGCCGAGAAAGTATCCATCATGGCTGA	ATTCGCCGCCAAGCTCTTCAGCAATATCAC	400

### Spontaneous differentiation and G418 selection

**Fig 1:**
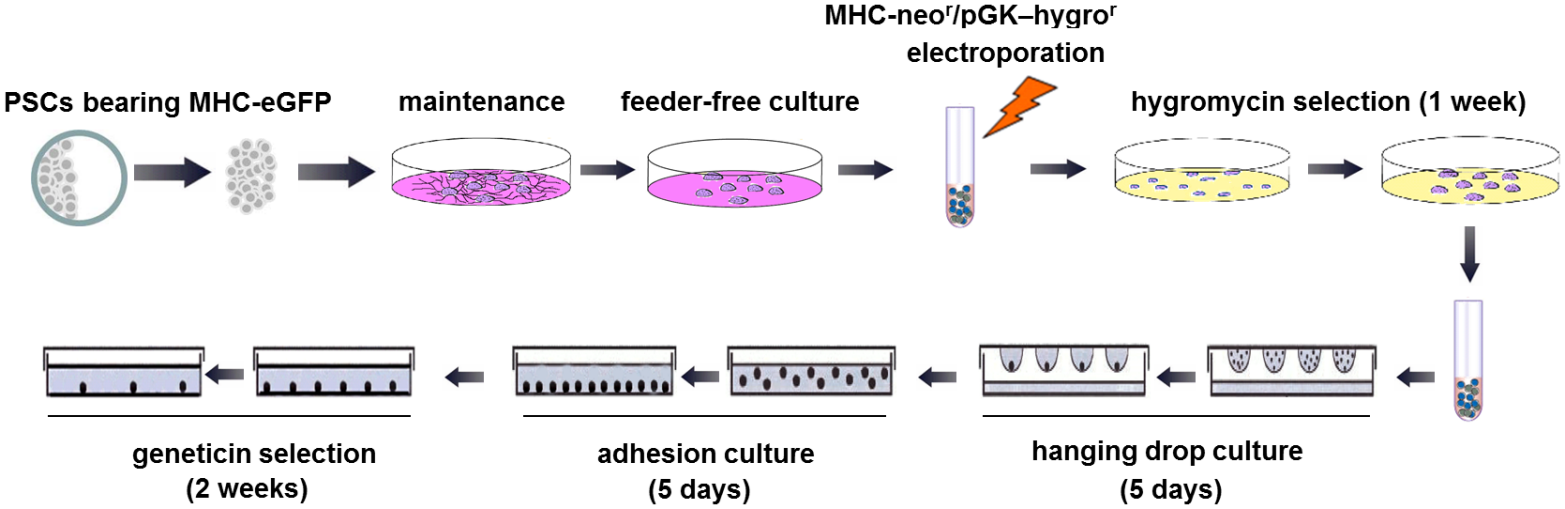
Schematic diagram of differentiation of parthenogenetic stem cells and cardiomyocyte enrichment.

To induce differentiation, PSCs were cultured in induction medium consisting of IMEM supplemented with 20% FBS, 1% NEAA, 2 mM L-glutamine, 0.1 mM β-mercaptoethanol and 0.5 mM ascorbic acid (Sigma). Hanging drop culture [12] was conducted for 5 days, followed by adherent culture for another 5 days (“5+5 process”; initiated by optimization of hanging drop input number in four individual experiments of 12 replicate studies). Subsequently, G418 selection was conducted at 250 μg/ml in the induction medium for 2 weeks (Fig 1). To further enhance drug selection, on day 13 (i.e. day 3 during G418 selection), the adherent colonies were incubated with 2 ml digestion solution containing IMDM supplemented with 0.2% collagenase type II (Sigma) and 60 U/ml DNase I (Sigma) at 37°C for 1 hour. The resulting cell clumps were collected and resuspended in 0.05% trypsin (Invitrogen) for further digestion for 5 min. Dissociated cells were then pelleted and replated into G418-supplemented induction medium in 0.1% gelatin-coated dishes. During detection of EFGP^+^ cell purity in a time-dependent manner, data were acquired in four individual experiments of 12 replicate studies.

### Cell transplantation

Animal care and all experimental procedures were performed in strict accordance with the Guide for the Care and Use of Laboratory Animals published by the US National Institutes of Health (publication No. 85–23, revised 1996). The handling of mice and all experimental procedures were specifically approved for this study by the Institutional Animal Care and Use Committee of Indiana University and Tongji University. For survival experiments, humane endpoints were applied to this study. For heart sample collection, all the mice were sacrificed by CO2 asphyxiation. All efforts were performed to minimize the distress and suffering of the animals. Animals were anesthetized with inhalation of 3% halothane and maintained on 1.5% halothane in 70% nitrous oxide and 30% oxygen. Cultures of PSC-derived cardiomyocytes were digested with trypsin, washed thrice with PBS, and resuspended in the pro-survival cocktail consisting of 50% (v/v) growth factor-reduced Matrigel (BD Biosciences, Bedford, MA, USA),100 mM ZVAD (benzyloxycarbonyl-Val-Ala-Asp(O-methyl)-fluoromethyl ketone; Calbiochem, San Diego, CA, USA), 50 nM Bcl-XL BH4 (cell-permeant TAT peptide, Calbiochem), 200 nM cyclosporine A (Novartis, Basel, Switzerland), 100 ng/ml IGF-1(Sigma) and 50 mM pinacidil (Sigma) [13]. Cells at 5 × 10^4^/recipient in 5 μl pro-survival cocktail solution were directly injected into the left ventricular free wall of anesthetized, intubated [C57Bl/6J x DBA/2J]F1 female siblings (with the age of 12 ± 1 weeks; weighing 28 ± 2 g; n = 23) using a 30-gauge tuberculin syringe. After extubation and evacuation of the pneumothorax, mice were placed at 37°C and monitored in micro-isolator cages (one per cage) until they recovered from surgery.

### Histology

Hearts were harvested, cryoprotected in 30% sucrose, embedded and sectioned at 10μm on a cryomicrotome as described previously [1]. Nonspecific immunoreactivity was blocked by incubation in 10% goat serum/1% bovine serum albumin in PBS. Sections were incubated with anti-α-actinin antibody (A7811, clone EA-53, produced in mouse; 1:100 dilution; Sigma) and immune reactivity was subsequently visualized using a rhodamine-conjugated secondary antibody (AP160R; 1:100; Millipore, Billerica, MA, USA). EGFP signal was visualized via epifluorescence.

### Imaging via 2-photon laser scanning fluorescent microscopy (TPLSM)

Images were recorded with an Olympus FV1000 Laser Scanning microscope. Illumination for 2-photon excitation was provided by a mode-locked Ti/Sapphire laser (Spectraphysics, Mountain View, CA, USA) and the excitation wave length was 810 nm. Hearts were imaged through a Nikon 1.2 numerical aperture water-immersion lens with a working distance of 200 μm. Emitted light was collected by 2 photomultiplier tubes fitted with narrow bandwidth filters for 560–650 nm and 500–550 nm, respectively. Images were collected at a resolution of 0.21 to 0.43 μm/pixel along the x–y-axis. For line-scan mode analysis, hearts were scanned repetitively along a line spanning EGFP^-^host and EGFP^+^ donor cardiomyocytes. Line-scan images were then constructed by stacking all lines vertically. Post-acquisition analysis was performed with MetaMorph software version 4.6r (Universal Imaging Incorporation, Downingtown, PA, USA). For determination of the time course of [Ca^2+^]_i_ decay, rhod-2 fluorescence intensity was normalized to the difference between peak and baseline intensity.

### Statistical analysis

Data are presented as mean ± standard deviation. Normality test, one way ANOVA and the SNK test were applied to pairwise comparison. The software SPSS 17.0 (SPSS Inc., Chicago, IL, USA) was used and *p*-values < 0.05 were considered statistically significant.

## Results

### Optimization of feeder-free conditions and electroporation voltage

**Fig 2:**
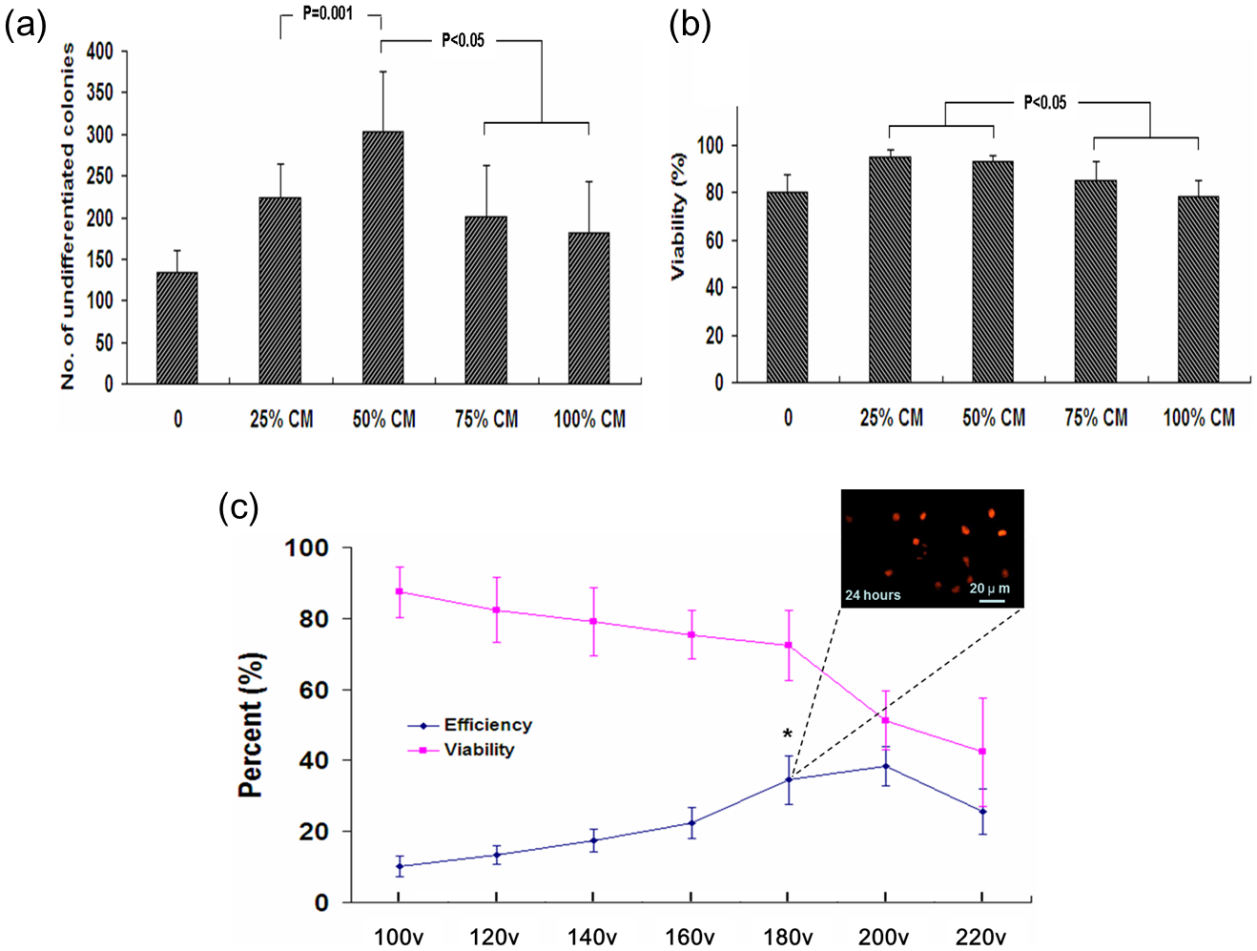
Characterization of growth and transfection efficiency of parthenogenetic stem cells (PSCs). (a, b) Effects of STO-conditioned medium (CM) on maintaining undifferentiated status (a; n = 12) and cell viability of PSCs (b; n = 12) after 2 days’ culture. (c) Viability and transfection efficiency of PSCs under electroporation at different voltages (n = 8). Results were evaluated according to ds-Red expressing reporter.

PSCs were grown onto STO cell feeder layers. As indicated, 50% STO-CM supplementation was shown to allow efficient maintenance of undifferentiated status of PSCs with highest cell viability (Fig 2a and 2b). Electroporation conditions were next optimized. PSCs were electroporated with a ubiquitously-expressed ds-Red reporter transgene, and transfection efficiency was calculated by the percent of ds-Red^+^ cells vs. the number of attached cells 24 h after electroporation. Electroporation at 180 V gave optimal results, with over 70% cell viability and over 30% transfection efficiency (Fig 2c). Higher voltage resulted in higher transfection efficiency at the cost of cell viability.

### Generation and characterization of MHC-neo^r^/pGK-hygro^r^ PSC cells

**Fig 3:**
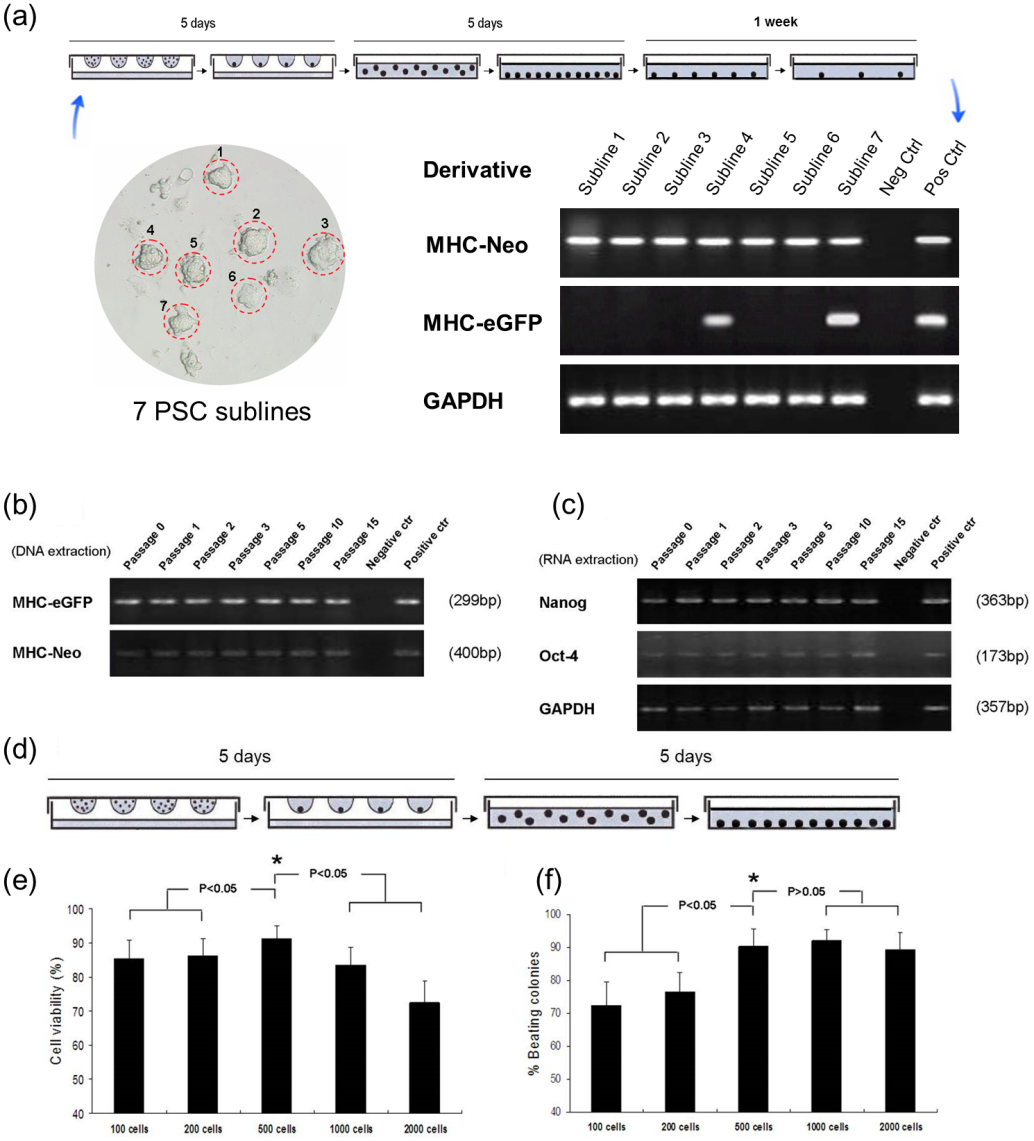
Transfection of PSCs with the MHC-neo^r^/pGK-hygro^r^ construct. (a) After hygromycin selection for 1 week, 7 MHC-neo^r^/pGK-hygro^r^ PSC sublines were generated. As indicated by PCR, PSC subline #4 and subline #7 exhibited stable integration of MHC-neo^r^ and MHC-EGFP in the genome. (b) Even after consecutive passages, PCR analysis showed stable integration of the MHC-neo^r^ and MHC-EGFP in the genome of PSC subline #4. (c) RT-PCR showed that PSC subline #4 retained robust expression of *Nanog* and *Oct4* transcript after passages, at levels similar to the parental cells. (d) Schematic diagram of the cardiac induction protocol. (e, f) Cell viability (e; n = 12) and frequency of cardiomyogenic differentiation (f; n = 12) in embryoid bodies (EBs) with initially variable cell input of PSC subline #4. The PSCs were cultured for 10 days (as illustrated in d, 5 days’ hanging drop culture followed by 5 days’ adhesive culture, also designated as “5+5 process”). * *p* < 0.05 vs. any other group.

Using conditions as optimized above, PSCs derived from the MHC-EGFP mice were transfected with the MHC-neo^r^/pGK-hygro^r^ construct. After hygromycin selection for 1 week, surviving colonies were picked for further propagation, and subject to serial passages. As expected, the sequences from the MHC-neo^r^/pGK-hygro^r^ were readily detected in the hygromycin-selected cultures (7 MHC-neo^r^/pGK-hygro^r^ PSC sublines were generated as illustrated in Fig 3a; data for subline #4 are shown in Fig 3b). The hygromycin selected clones exhibited robust expression of Oct-4 and Nanog (Fig 3c). PSC differentiation was induced in hanging drop cultures (Fig 3d). Input cell content was optimized in terms of cell viability and frequency of cardiomyogenic differentiation (as evidenced by the presence of beating colonies) at day 10; an input of 500 cells per drop was optimal (Fig 3e and 3f).

### Genetic selection of PSC-derived cardiomyocytes

**Fig 4:**
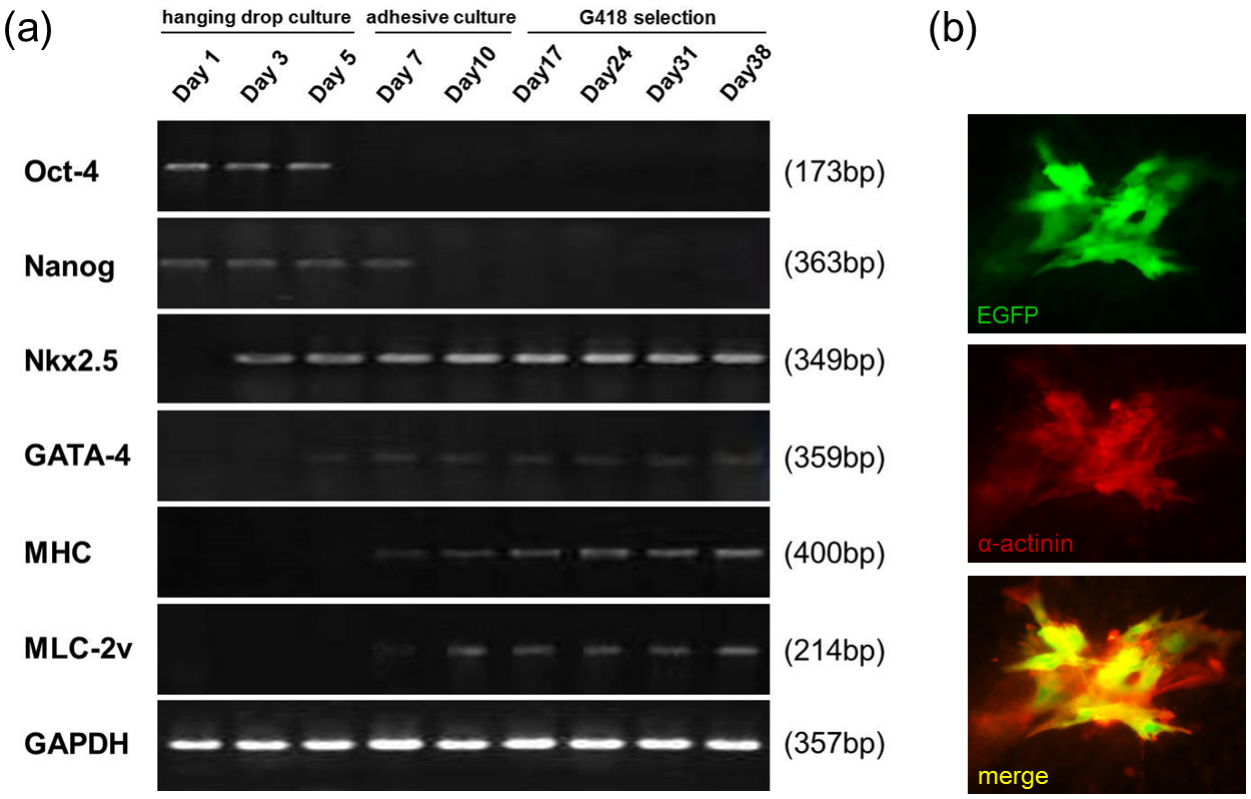
Cardiomyogenic differentiation and selection of PSCs. (a) RT-PCR analysis for stemness markers (Oct-4 and Nanog) and cardiac lineage-specific markers (Nkx2.5, GATA-4, MHC, MLC-2v) through the differentiation and selection processes. (b) Co-localization of EGFP and α-actinin immune reactivity confirmed the cardiomyogenic identity of derived EGFP^+^ cells (at day 17).

**Fig 5:**
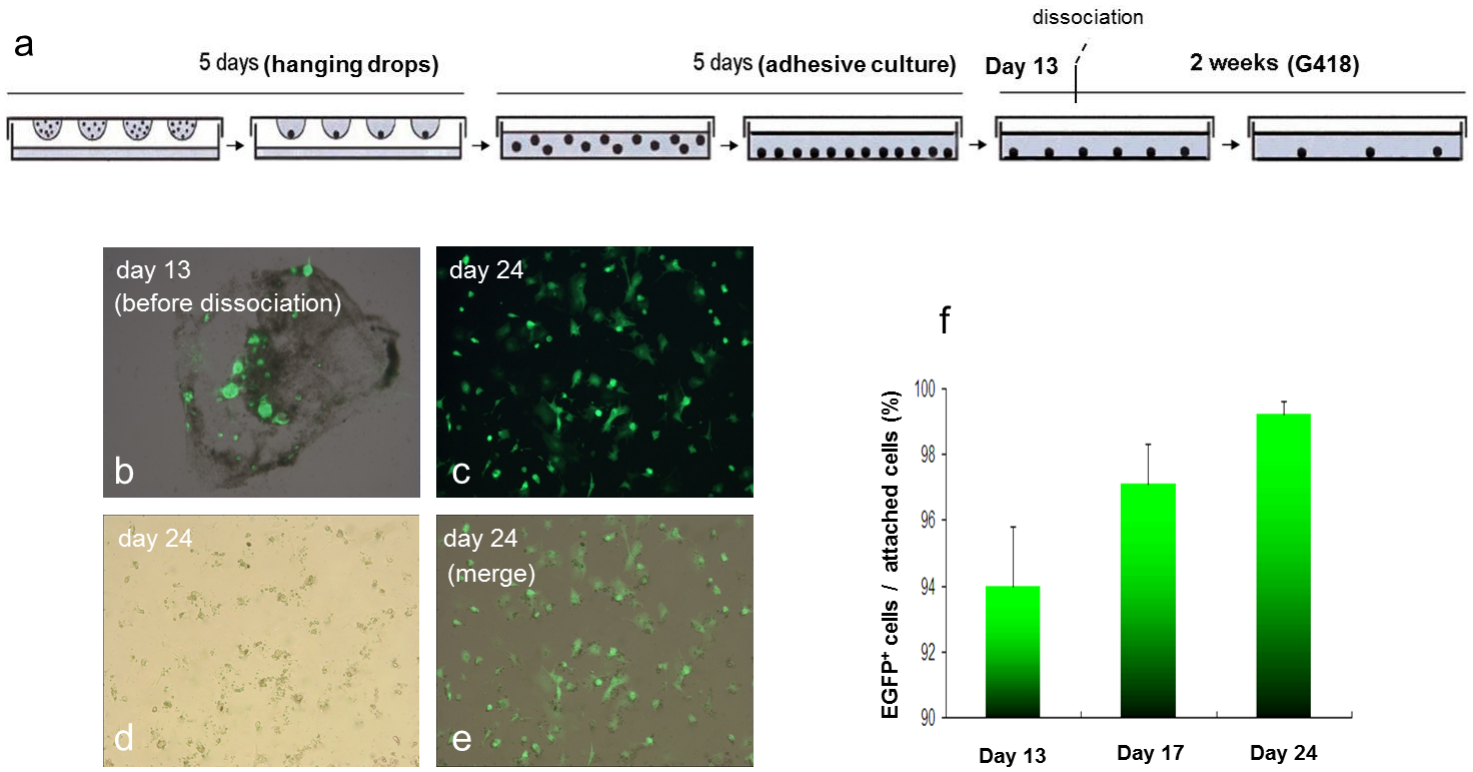
Cardiomyocyte enrichment. (a) Schematic diagram of enrichment process; (b-e) Progressive enrichment of EGFP^+^ cardiomyocytes over time (as indicated) with G418 selection. (f) Purity of EGFP^+^ cardiomyocytes at day 13, day 17 and day 24 during G418 selection (n = 12).

PSC subline #4 was cultured in hanging drops for 5 days, followed by adherent culture for another 5 days. G418 selection was then imposed. RT-PCR analyses revealed a progressive decrease in Oct-4 and Nanog expression, consistent with the differentiation of the cells (Fig 4a). A concomitant increase in the expression of the cardiomyogenic lineage determining genes Nkx2.5 and GATA4, followed by expression of the sarcomeric protein genes MHC and MLC-2v, was also apparent. Co-induction of EGFP epifluorescence (from the MHC-EGFP reporter) and α-actinin immune reactivity was readily apparent (Fig 4b). Expression of the MHC-EGFP reporter was used to quantitate cardiomyocyte content following G418 selection. Cultures of selected cardiomyocytes were dispersed into individual cells and replated in the presence of G418 (Fig 5a). A progressive increase in cardiomyocyte content was apparent (Fig 5b–5f).

### Engrafted PSC-derived cardiomyocytes obtained via genetic selection form a functional syncytium with the host myocardium

**Fig 6:**
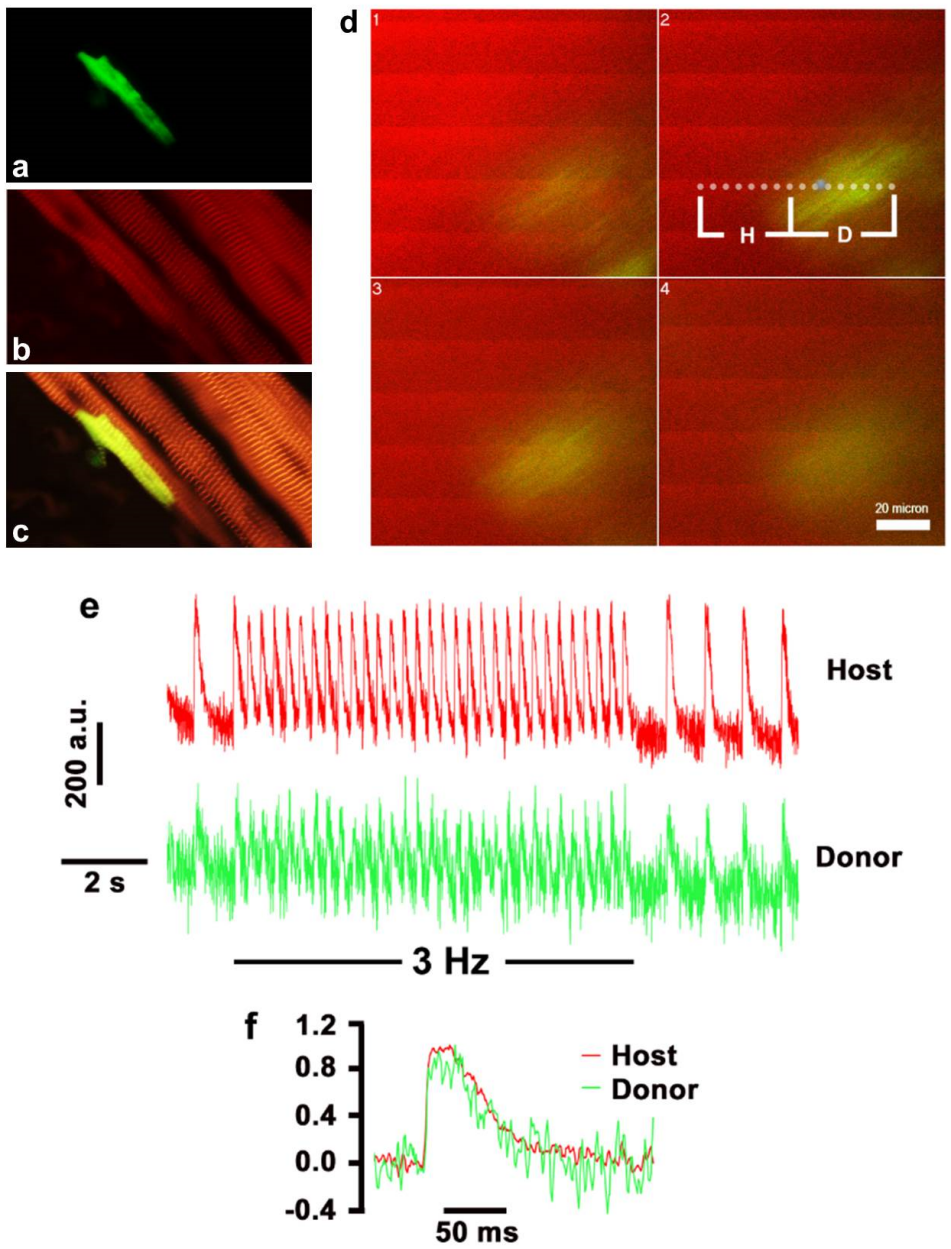
G418-selected PSC-derived cardiomyocytes are functionally integrated with the host myocardium. (a) The presence of EGFP^+^ PSC-derived cardiomyocyte in the host heart at 2 weeks post transplant. (b) α-actinin immunofluorescence of the same donor cell as in (a). (c) Merge of (a) and (b). (d-f) Two-photon laser scanning microscopy revealed synchronous action potential-evoked calcium transients in the EGFP^+^ donor cell and its adjacent EGFP^-^host cardiomyocytes. (d) 2-D images taken at 16-μm *z*-steps across an EGFP^+^ donor (D) myocyte and its surrounding host (H) myocytes. The heart was being paced at a frequency of 3 Hz during image acquisition. Periodic increases in rhod-2 fluoresence intensity reflect increases in cytosolic calcium in response to propagating action potentials and span the entire width of the image, including the EGFP^+^ donor cell. The dotted line denotes the position of the scan line for line-scan imaging. (e) Line average as a function of time for the portions of the dotted scan line indicated by brackets in (d). Line scans were obtained during spontaneous sinus rhythm and during electrical pacing at 3 Hz. Action potential-evoked calcium transients in the host myocyte are in phase with those in the neighboring host myocytes. (f) Superimposition of normalized calcium transients for the donor and host myocytes, demonstrating similar kinetics of the transients in both cell types.

PSC-derived cardiomyocytes obtained by G418 selection were injected into the left ventricular free wall of [C57Bl/6J x DBA/2J]F1 mice (the genetic background of the mice originally used for PSC derivation). Hearts were harvested two weeks later, and processed for immune histologic analysis. Donor cardiomyocytes were readily detected by EGFP epifluorescence, and exhibit a typical rod-shaped morphology (Fig 6a). Anti-α-actinin immune fluorescence indicated that the donor cardiomyocytes had well developed myofibers which were aligned in parallel with those in the host cardiomyocytes (Fig 6b and 6c). TPLSM revealed the presence of synchronous action potential-evoked calcium transients in the EGFP^+^ donor cell and its adjacent EGFP^-^host cardiomyocytes (Fig 6d–6f). Line averages as a function of time for a donor-derived, EGFP-expressing myocyte and their neighboring, non-expressing host cardiomyocytes revealed that the calcium transients in the donor cell were entrained by the electrical activity of the recipient myocardium both during sinus rhythm and during electrical pacing at 3 Hz (Fig 6e). Further, the calcium transient kinetics in the donor and host myocyte was indistinguishable from each other (Fig 6f). Overall, these results suggest that the transplanted PSC-derived cardiomyocytes were structurally and functionally mature, and were electrically coupled with the host cardiomyocytes.

## Discussion

We have previously shown that PSCs derived from mice carrying an MHC-EGFP reporter transgene can differentiate into cardiomyocytes *in vitro* and *in vivo*, and furthermore that PSC-derived cardiomyocytes isolated via directed differentiation could be functionally engrafted into recipient hearts [8]. In this study, we extend these observations and show that genetically modified PSCs could be easily generated and used to produce essentially pure populations of cardiomyocytes via simple G418 selection of spontaneously differentiating cultures. Moreover, the selected PSC-derived cardiomyocytes functionally integrate with the host myocardium following engraftment.

Many forms of cardiac disease have as a common endpoint, i.e., cardiomyocyte loss, and therapeutically restoring cardiac mass is likely to be beneficial. The notion of cell transplantation for myocardial repair was initially introduced during the early 1990s, and donor cells from these early studies included bona fide cardiomyocytes [1,14], ESC-derived cardiomyocytes [5,15], and a plethora of adult-derived stem cells with varying degrees of cardiomyogenic activity [16]. Alternative strategies to restore cardiac mass in damaged hearts include induction of cardiomyocyte proliferation [17–19], as well as directed trans-differentiation of resident non-myocytes into cardiomyocytes [20,21]. Each approach has potential benefits and limitations, with regards to efficacy and potential adverse side effects.

With regard to cell transplantation, many claims of adult-derived stem cells forming transmural grafts have failed to be replicated by others [22–26]. While some controversy remains in this field, the absence of reproducible overt cardiomyogenic activity raises concerns regarding the robustness of these cells to form large numbers of replacement cardiomyocytes. Thus while these cells could impart benefit upon transplantation [27], it almost certainly arises independently of donor cell cardiomyogenic activity. In light of this, transplantation of bona fide cardiomyocytes, derived from ESCs or ESC-like progenitors (i.e., iPSCs or PSCs) constitutes the currently best validated source of donor cells which can restore muscle mass. With the notion of allogenic application, PSCs have the distinct advantage that a comparatively small panel of cells should give rise to donor cardiomyocytes which would be immunologically tolerated for the vast majority of potential recipients, making PSCs a very attractive source for further development.

The ability of PSCs to be easily modified genetically, and furthermore their ability to give rise to highly enriched cardiomyocyte cultures capable of functional engraftments are an important observation. Indeed, a recent proof-of-concept study demonstrated functional engraftment of human ESC-derived cardiomyocytes into immune suppressed monkeys with small cardiac infarcts [28]. These studies required further efforts to generate sufficient numbers of donor cells for engraftment using directed differentiation approaches [29]. In contrast, mouse ESCs carrying the MHC-neo selection cassette gave rise to large numbers of cardiomyocytes using bioreactors [9,10]. Reproducible, economical and relatively fast large-scale generation of donor cells will be an important consideration for potential therapeutic applications.

In our previous studies, we have confirmed similar fundamental properties in murine PSCs and ESCs, despite notable differences in allelic variability and differential imprinting characteristics. Haploidentity of major histocompatibility complex in PSCs is particularly attractive for allogeneic cell-based therapies and we have confirmed acceptance of PSCs in major histocompatibility complex-matched allotransplantation (8). The data presented here builds on our previous studies with murine PSCs, and demonstrates their genetic tractability, as well as the ability to generate pure populations of cardiomyocytes using a relatively simple selection protocol with the transgenes of MHC-neo^r^/pGK-hygro^r^ and MHC-EGFP. The utility of any source of donor cardiomyocytes for cardiac repair will ultimately depend on their ability to effect replacement of large areas of damaged myocardium. Future efforts will be directed towards that end.

## Supporting Information

S1 File The completed form for Animal Research: Reporting In Vivo Experiments. The ARRIVE Guidelines Checklist.
(DOCX)
