# Supplementary material for: Cardiac Engraftment of Genetically-Selected Parthenogenetic Stem Cell-Derived Cardiomyocytes
Source: PLoS One. 2015 Jun 25;10(6):e0131511. doi: 10.1371/journal.pone.0131511 (PMC4482509; doi:10.1371/journal.pone.0131511)

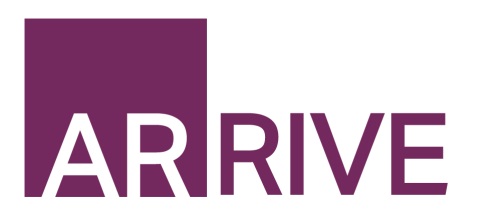


The ARRIVE Guidelines Checklist

Animal Research: Reporting In Vivo Experiments

Carol Kilkenny^1^, William J Browne^2^, Innes C Cuthill^3^, Michael Emerson^4^ and Douglas G Altman^5^

*^1^The National Centre for the Replacement, Refinement and Reduction of Animals in Research, London, UK, ^2^School of Veterinary Science, University of Bristol, Bristol, UK, ^3^School of Biological Sciences, University of Bristol, Bristol, UK, ^4^National Heart and Lung Institute, Imperial College London, UK, ^5^Centre for Statistics in Medicine, University of Oxford, Oxford, UK.*

|  | | ITEM | RECOMMENDATION | Section/ Paragraph |
| --- | --- | --- | --- | --- |
| 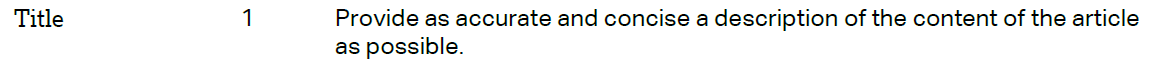 | | | Title |  |
| 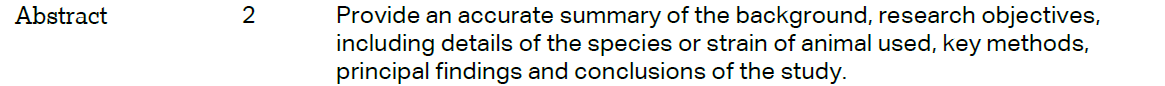 | | | Abstract |  |
| INTRODUCTION | | |  |  |
| 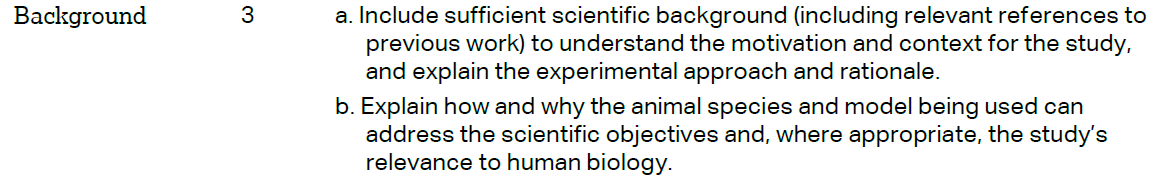 | | | Paragraphs 1-2  Paragraphs 2-3 |  |
| 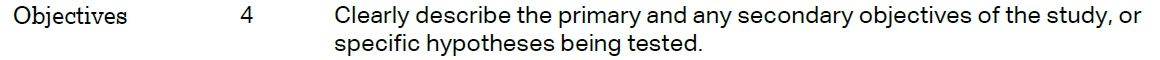 | | | Paragraph 3 |  |
| METHODS | | |  |  |
| 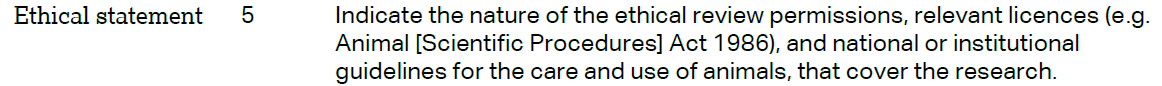 | | | Paragraph 5 |  |
| 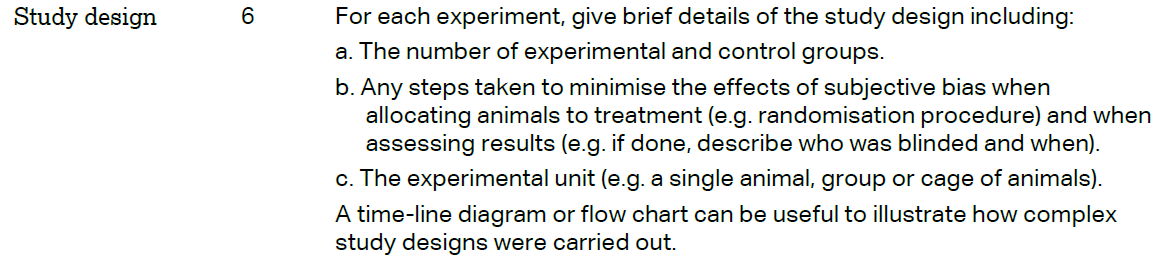 | | | Paragraphs 1, 2 & 4  Paragraph 6  Paragraph 5  Figure 1 |  |
| 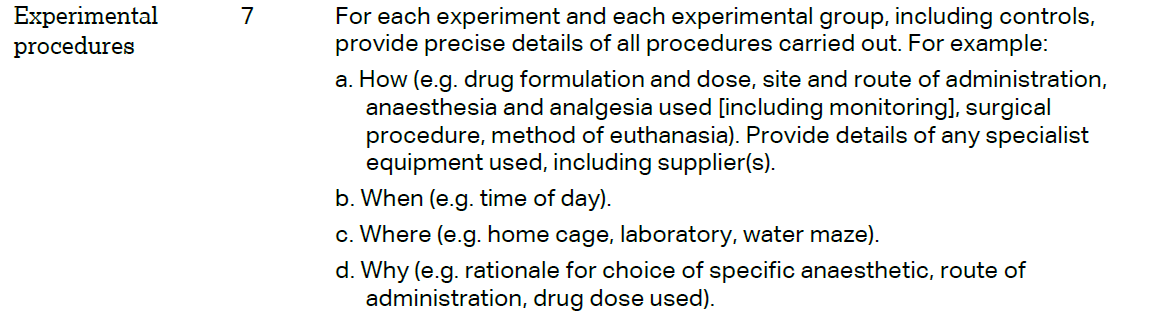 | | | Paragraphs 5 & 7 |  |
| 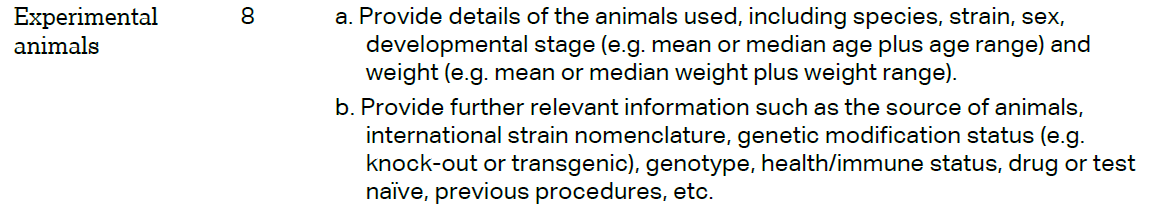 | | | Paragraph 5  Paragraphs 1 & 5 |  |

The ARRIVE guidelines. Originally published in *PLoS Biology*, June 2010^1^

| 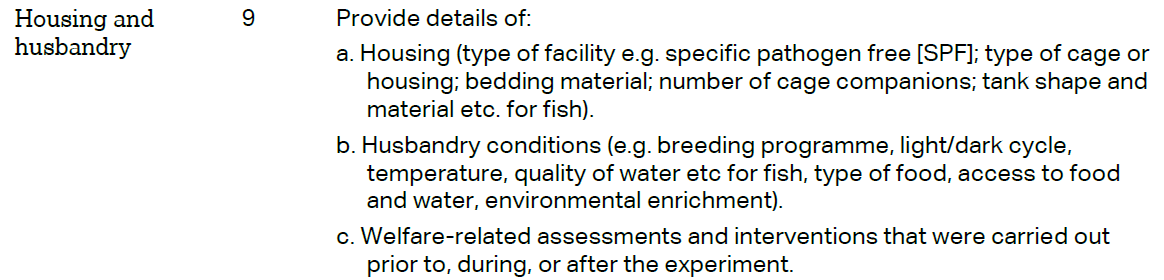 | Paragraph 5 | |
| --- | --- | --- |
| 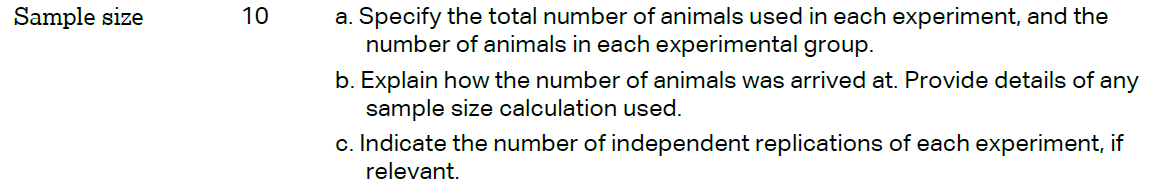 | Paragraph 5 | |
| 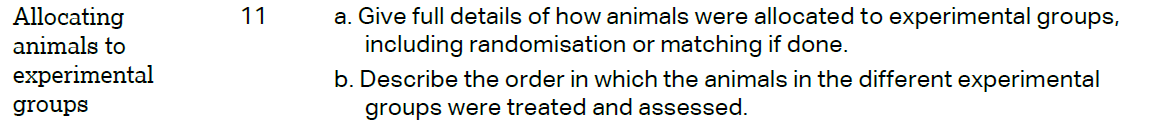 | Paragraphs 1 & 5 | |
| 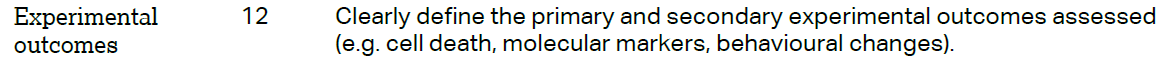 | Paragraphs 6 & 7 | |
| 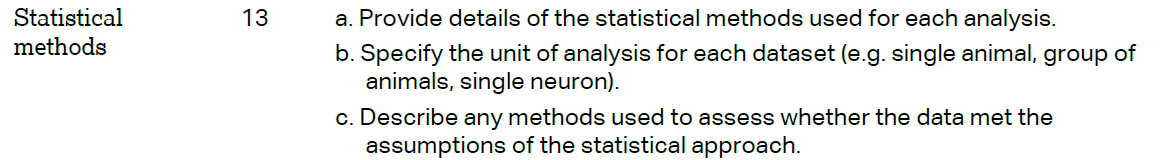 | Paragraph 8 | |
| RESULTS |  | |
| 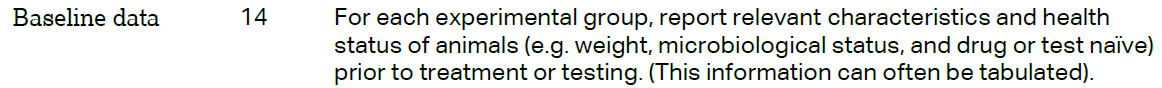 | Methods  Paragraph 5 | |
| 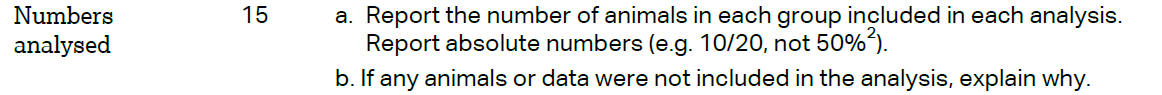 | Methods  Paragraph 5 | |
| 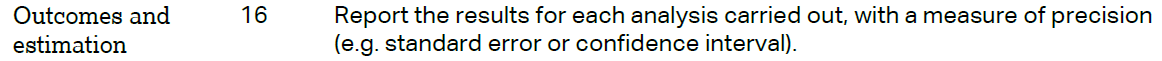 | Paragraphs 1, 2 & 4; Figures 2, 3 & 5 | |
| 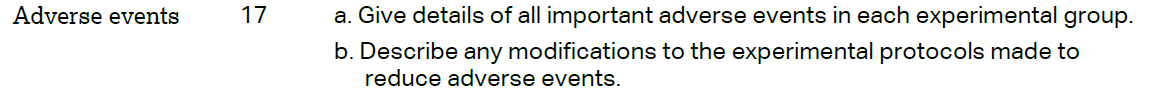 | Paragraph 4 | |
| DISCUSSION |  | |
| 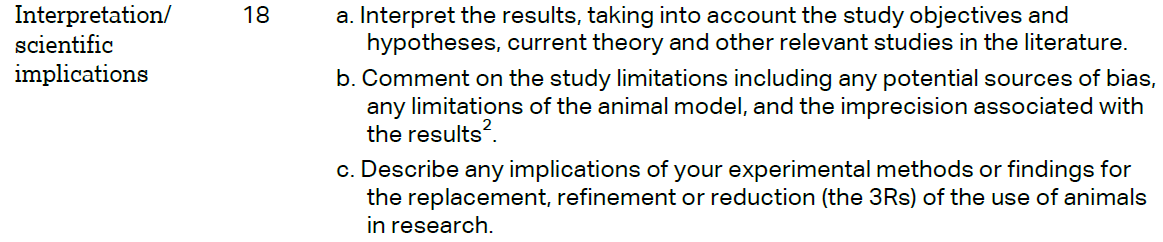 | Paragraphs 1-4  Paragraph 5  Paragraph 5 | |
| 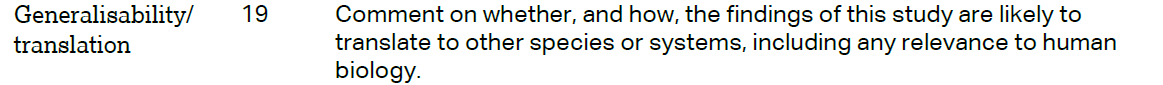 | Paragraphs 3, 4 & 5 | |
| 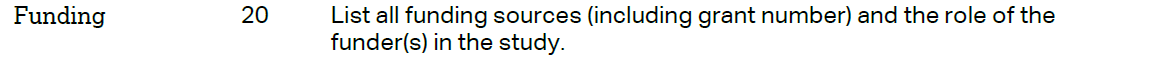 | | As indicated during submission |


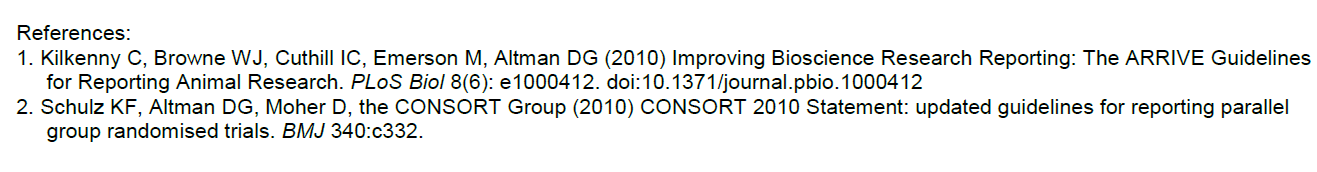

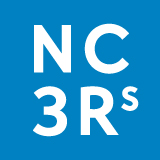

Supplement: S1 File — The ARRIVE Guidelines Checklist. (DOCX) [file pone.0131511.s001.docx]
